# East with the Night: Longitudinal Migration of the Orinoco Goose (*Neochen jubata*) between Manú National Park, Peru and the Llanos de Moxos, Bolivia

**DOI:** 10.1371/journal.pone.0046886

**Published:** 2012-10-04

**Authors:** Lisa C. Davenport, Inés Nole Bazán, Nancy Carlos Erazo

**Affiliations:** 1 Duke University Center for Tropical Conservation, Durham, North Carolina, United States of America; 2 Universidad Nacional Mayor de San Marcos, Lima, Peru; 3 Universidad Peruana Cayetano Heredia, Lima, Peru; Institut Pluridisciplinaire Hubert Curien, France

## Abstract

We report on the intra-Amazonian migration of a pair of Orinoco Geese (*Neochen jubata*) from Manú National Park, Peru. The species is Critically Endangered in Peru, so a major aim of the study was to aid conservation planning by learning the wet season location of the country's last known breeding population. We captured a breeding pair on October 27, 2010, and fitted the birds with Microwave Telemetry, Inc. GPS/Argos satellite PTT's. The pair migrated ∼655 km from Manú National Park to the Llanos de Moxos, Bolivia (Dept. of Bení) in a predominantly longitudinal migration, reaching their final destination on December 23, 2010. Major movements (>5 km per time period) were almost exclusively at night and were undertaken with and without moonlight. Foraging areas used at stopovers in the Llanos de Moxos were remarkably limited, suggesting the importance of grazing lawns maintained by the geese and other herbivores, possibly including cattle. Orinoco Geese are resident in the Llanos de Moxos year-round, so the Manú geese represent a partial migration from the Bení region. We hypothesize that cavity nest limitation explains the partial migration of Orinoco Geese from the Llanos de Moxos.

## Introduction

Bird migration studies have generally focused on north-temperate species. Considerably less well-studied are austral migrations within southern latitudes [1] and the least understood of all are intra-tropical migrations [2]. At our fieldsite in Manú National Park, approximately 12% (62/526) of birds are observed to undergo seasonal movements/migrations, of which perhaps 5% (25) are suspected to be intra-tropical migrants or regional wanderers (J. Terborgh, pers. obs.); the Orinoco Goose (*Neochen jubata*) is the first of these species to be tracked.

A motivation for this study was concern for the Orinoco Goose in Peru, where it is listed by IUCN as Critically Threatened; the only known breeding population in Peru is in Manú, comprising fewer than 30 breeding pairs. The species was probably once common throughout Amazonian Peru, having been reported as common on dry season beaches in the Department of Loreto in 1768 by Graz, a Jesuit priest [3]. Most populations appear to be in decline excepting possibly those of Bení, Bolivia, and the Araguaia River, Brazil [4].

Orinoco Geese are members of the shelduck subfamily Tadorninae. They are secondary cavity nesters, using the palm *Iriartea deltoidea* (Venezuela) and other trees [4]. In Manú, Orinoco Geese breed in the dry season (May-Oct), but disappear each wet season (Nov-Apr). Prior to this study, it was not known where the birds migrated in the wet season.

## Methods

### Ethics Statement

This study was approved by the Institutional Animal Care and Use Committee of Duke University (Protocol #A081-10-03) and by the Peruvian National Parks Service (SERNANP) under permit # 09-S/C –2010 SERNANP-PNM. The weight of the PTT's and Teflon harness was around 2% of the birds' weights (by sex), below the 3% recommended as best practice [5].

**Figure 1:**
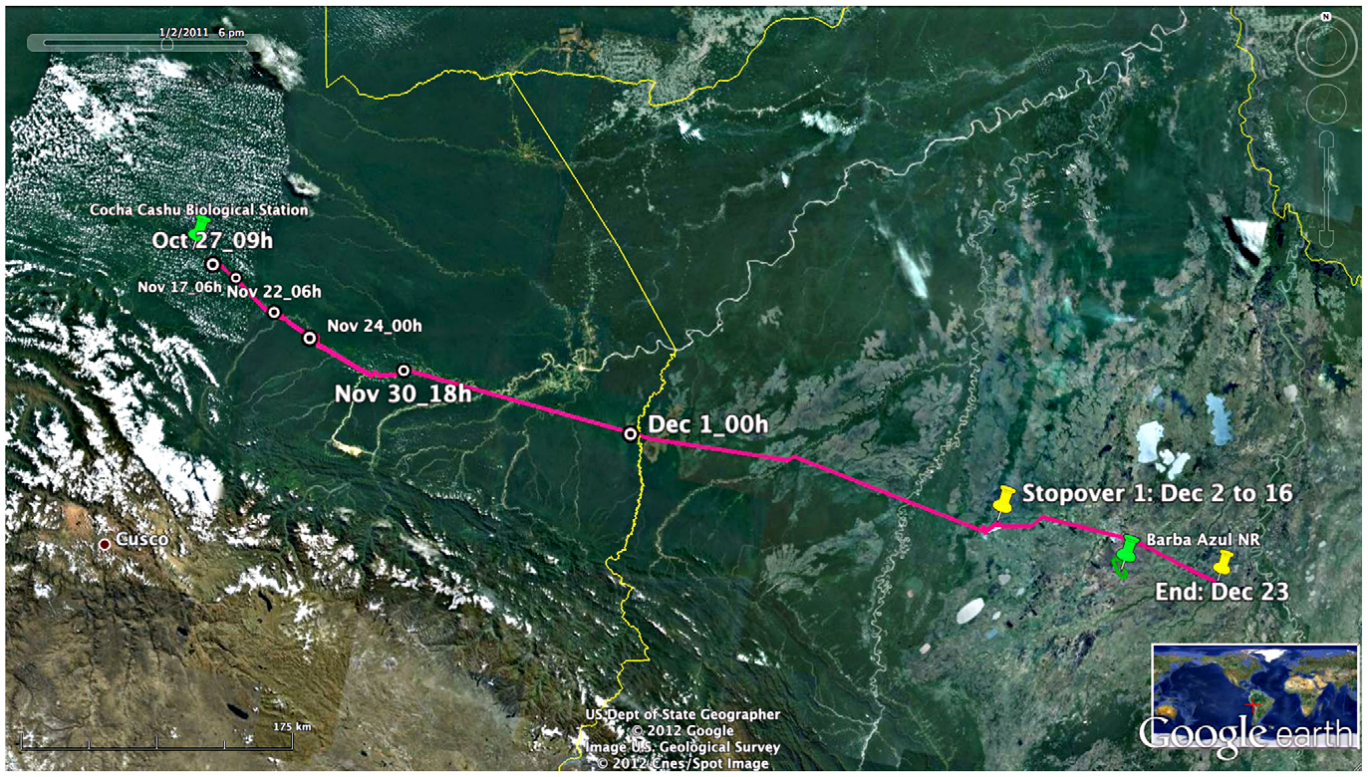
Orinoco Goose Migration Route. The complete migration (pink line) of the Orinoco Geese caught in Manú National Park, October 27, 2010. Locations denote date and local time for points discussed in the text. Google Earth accessed 19 April 2012 coordinates 13°14′33.19′′S; 68°35′31.77′′W.

### Study Sites

The Río Manú in Manú National Park, Peru, situated in tropical moist forest [6] with an elevation of 320 m at the trapping location. Beginning about July, Manú beaches experience flushes of new vegetation whose leaves and seeds are consumed by waterfowl such as Orinoco Geese, Muscovy Ducks (*Cairina moschata*) and Horned Screamers (*Anhima cornuta*). Some typical beach plants include *Amaranthus spinosus*, *Eclipta alba*, *Eleusine indica* (a favorite food plant of Orinoco Goose, particularly the seed heads – pers. obs. LCD), *Ludwigia spp., Panicum sp., Solanum americanum, Tessaria integrifolia*, and *Torulinium odoratum*. Large swaths of exposed beaches are typically covered by seedlings of *T. integrifolia*, although this plant does not appear to be a preferred food plant of Orinoco Geese or other waterfowl.

**Figure 2:**
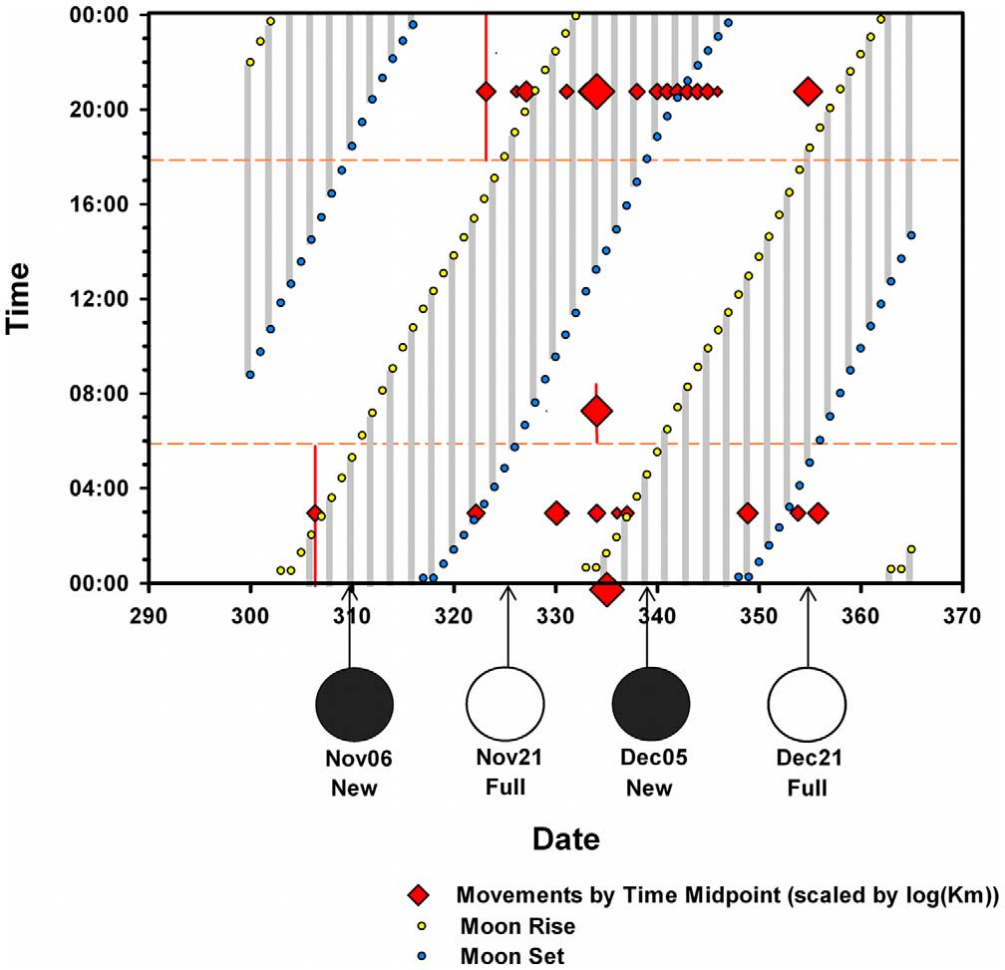
Major Movements by Date, Time and Phase of the Moon. Major Movements (>5 km per period) by Julian date, time, and phase of the moon between October 27 and December 31, 2010. For each day, shaded areas are times when the moon is not visible in the sky at the goose's location. Movements are diagrammed by date and time with red diamonds sized in relation to the log of the distance traveled (in km) during that period. Each movement is placed at the midpoint of the time period during which the goose traveled, which is typically within a 6 h period between 6 pm and midnight or midnight and 6 am. Vertical red lines on the furthest left points and one anomalous point at 07∶00 demonstrate these potential spreads in timing.

The Llanos de Moxos, in the Dept. of Bení, Bolivia are low elevation (100–200 m), seasonally-flooded savannas, interspersed with gallery forests and elevated levees [7]. Waterfowl are prolific in the area, including Orinoco Goose, White-faced and Black-bellied Whistling Ducks (*Dendrocygna viduata*, and *Dendrocygna autumnalis*), Muscovy Duck *(Cairina moschata)*, Brazilian Teal (*Amazonetta brasiliensis*), and a number of herons, ibises, storks, and Roseate Spoonbills (*Ajaia ajaja*), among others. Elevated forest islands where geese and parrots nest are dominated by the palm *Acrocomia aculeata* (“Totai” – M. Herrera, pers. comm.).

### Capture and Harnessing

We captured a pair of adult geese on a Río Manú beach (coordinates 11.96° S, 71.29° W) below Cocha Cashu Biological Station on October 27, 2010, using a compressed-air cannon (Waterfowl “Net-Blaster”, Wildlife Control Supplies, Inc.) that deployed a 40′ x 60′ net of 1 ¼′′ mesh constructed of Diamond Braid #147 Nylon. We attracted wild birds to the trapping zone with live decoys placed in a cage in the centre of the net's reach. Decoys were pet Orinoco Geese kept by Matsigenka villagers. Observers activated a remote switch to trigger the cannon net when wild geese were within the trapping zone.

**Figure 3:**
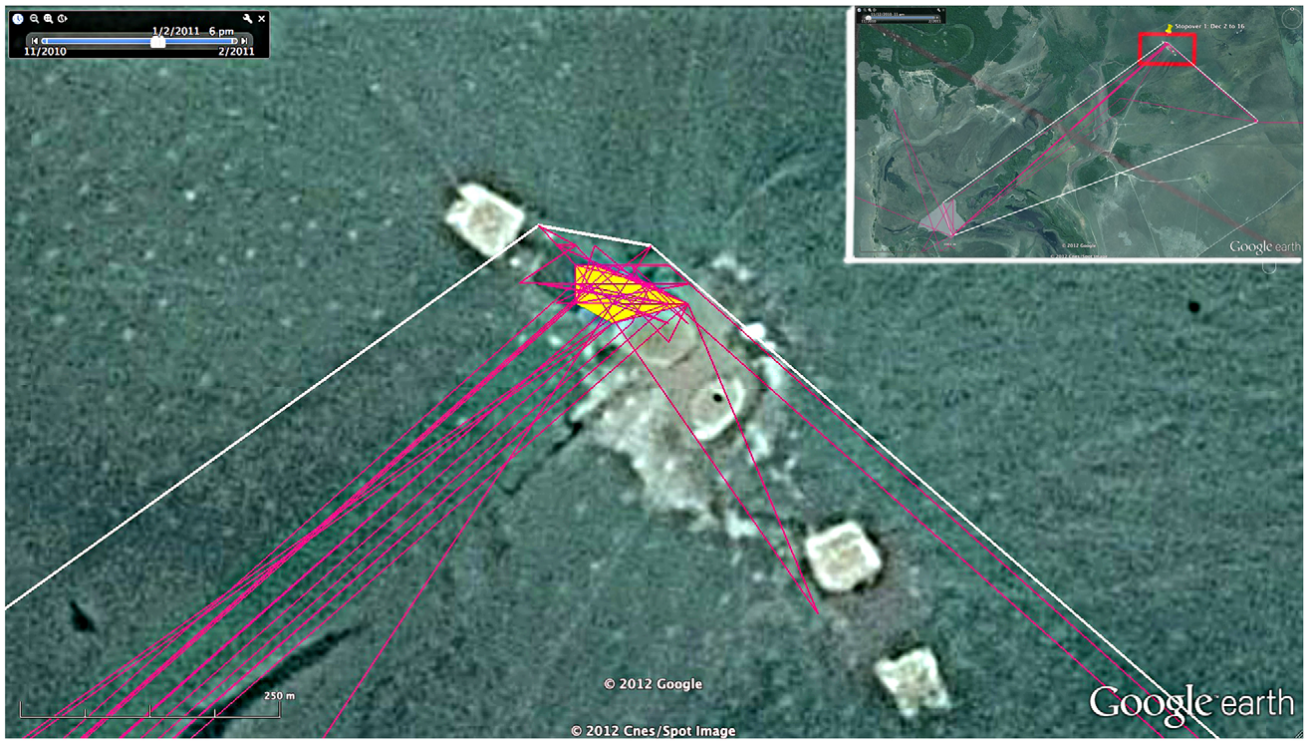
Stopover 1 50% MCP Core Foraging Area. Daytime foraging area used by the Orinoco Geese at Stopover 1, December 2 to December 16. Inset map shows the foraging location with respect to the typical night-time roost about 10 km away and 95% Minimum Convex Polygon (MCP) of daytime and night-time locations. The underlying yellow polygon is the 50% MCP core area of use. Google Earth accessed 11 April 2012 coordinates 13°28′31.23′′S; 66°49′28.44′′W.

Birds captured were fitted with Microwave Telemetry, Inc. (MTI) GPS/ARGOS PTT 100′s attached with backpacks of 0.33 inch Teflon ribbon (Bally Ribbon Mills). The backpacks were custom-fit to each bird, using a knotting protocol generally following [8].

Geese can be highly destructive to backpack harnesses; they may also preen feathers over a unit's solar panels, causing power loss [9]. We therefore used two different PTT's: one solar and one battery-operated. The larger male carried a 40-g battery-powered PTT that provided a single daily GPS location at noon local time. The female carried a 30-g solar-powered PTT that provided multiple daytime locations plus one location at midnight local time. The female's PTT was programmed to provide fixes every 2 hours during daylight hours beginning at 06∶00, and one fix at midnight (00∶00) during the expected migration period (November 1 to January 1) and every 3 hours (beginning at 06∶00) plus one midnight fix following the expected migration period.

**Figure 4:**
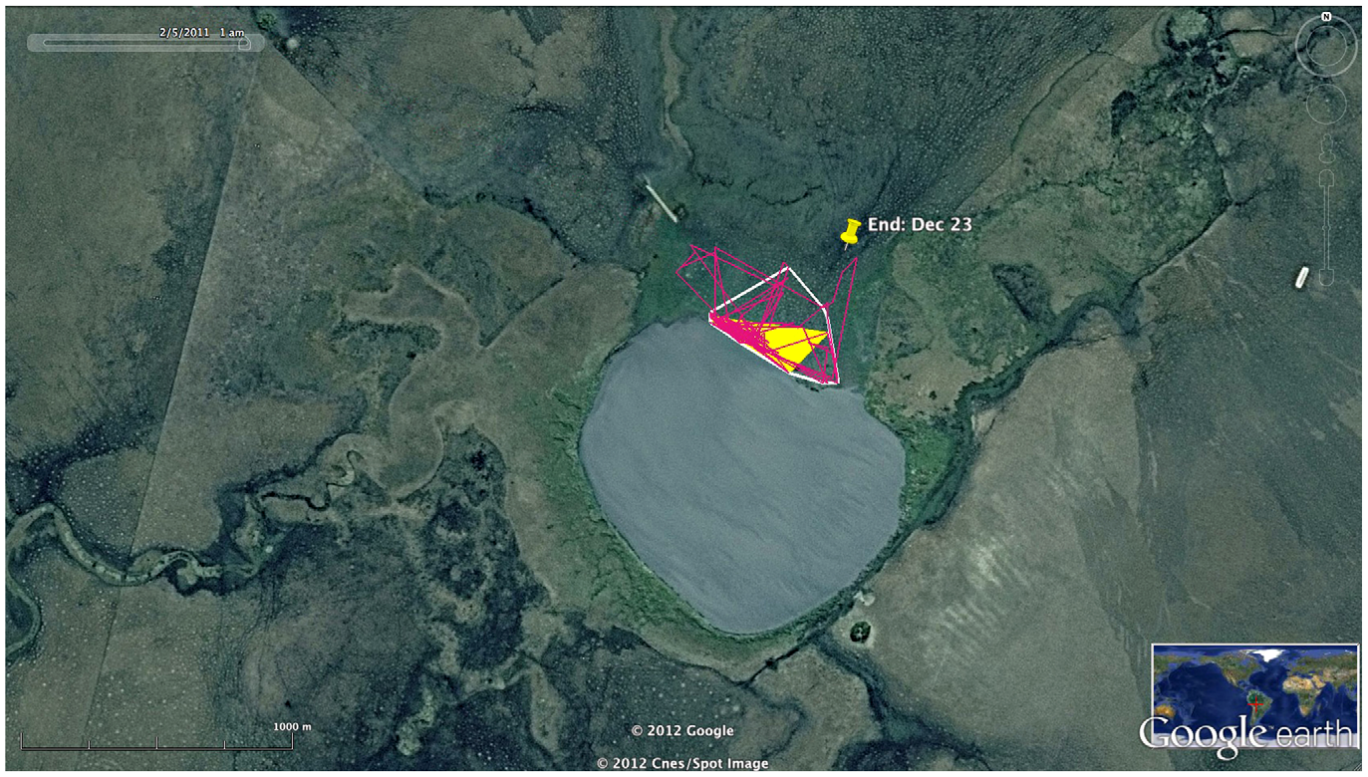
Final Destination Foraging Area. Foraging area used by the Orinoco Geese at their final destination, December 23 to Feburary 4. The white polygon is the 95% Minimum Convex Polygon (MCP), and the underlying yellow polygon is the 50% MCP core area of use. Google Earth accessed 11 April 2012 coordinates 13°9′39.56′′S; 65°32′33.07′′W.

### Data Preparation and Analysis

Location data were downloaded from the ARGOS satellite system, parsed using the MTI Argos-GPS Parsing software (v. 2010Jul15), and mapped using Biotas v. 1.03 and Google Earth (v. 6.1.0.5001). The full dataset is archived at www.movebank.org [10] and available for public view. Because the pair migrated together to their final destination, for simplicity, only the female's data are included in analyses and figures.

We used Biotas v. 1.03 to calculate 95% and 50% Minimum Convex Polygons (MCP) for subsets of the female's daily fixes, including the stopover location used December 2 to December 16 (“Stopover 1” in Figure 1) and the final destination (“End” in Figure 1). MCP was used because some subsets of the data had too few points to compute Kernel Density estimates. At Stopover 1, night-time roosts were not contiguous with daytime feeding locations, so we calculated the 95% MCP for day and night-time points separately. At the final destination, day and night-time roosts were contiguous, so we calculated the 95% MCP and 50% MCP of all points.

Finally, we calculated great circle distance between all of the female's locations for daytime (06∶00 to 18∶00) vs. night-time (18∶00 to 06∶00) movements, and plotted the date and time of all major movements (>5 km in a time period) with the time of moonrise and moonset at that date and location (Figure 2). The moon's schedule was obtained for each date/location from the Garmin Mapsource (6.13.7) Celestial Information Utility. Distance between daily points in phase 1 were calculated for points taken at 06∶00 daily between October 27 and November 22, the date when the geese left the park, and also when major moves became increasingly common.

## Results

The pair of Orinoco Geese migrated ∼655 km from Manú National Park, Peru, to the Llanos de Moxos, Bolivia (Dept. of Bení) in two distinct phases (Figure 1). In the first phase, the geese slowly descended the Río Manú and the Río Madre de Dios, stopping on river beaches over the course of a month, to November 30, 2010. Average distance between daily points was 3.7 km in this phase (±6.2 km SD). In the second phase, upon reaching the confluence of the Río Madre de Dios and Río de los Amigos, the geese flew overland (heading of 106°) to lakes of the Llanos de Moxos. About 60% of their total migration was undertaken over the first two days of this phase (November 30 to December 2) after which they spent a few weeks at various intermediary points before reaching their final destination on December 23.

Unlike most bird migrations, the Orinoco Goose migration constituted a far greater shift in longitude than latitude (Δ long  = 5.7° east; Δ lat  = 1.9° south). The pair migrated together, except for a 5-day separation around their first stopover. All but one major move occurred at night, and these included both moonlit and moonless periods (Figure 2).

The pair spent about two weeks (Dec 2 to 16) at one intermediary stopover lake (“Stopover 1: Dec 2 to 16” in Figure 1). The only other stopovers were of short duration (1–4 days) before completing their migration upon arriving at a lake west of the Rio Mamoré (“End: Dec 23” in Figure 1; coordinates 13.83° S, 65.54° W).

Local movements around Stopover 1 and at the final destination were remarkably limited in the area used for foraging. Around Stopover 1, daytime foraging occurred in a 0.4 ha area in a notably disturbed area, possibly cattle ponds (Figure 3). Each night, the birds moved 9.6 km to a roosting site along a lakeshore (95% MCP for night-time points 0.7 ha).

At the final destination, the geese used a night-time roost contiguous with daytime feeding sites along a lakeshore. Combining all points, their movments covered a 95% MCP area of only 11.3 ha (50% MCP of 3.9 ha) between December 23 and February 4 when the female began a return migration along her outbound path (Figure 4).

The male's transmitter went silent on December 31, after providing 65 unique GPS fixes, whereas the female continued transmitting through February 17 (677 unique GPS fixes). Between February 4 and the female's last new location on February 17, she returned to some of the same stopover points she used on the outbound migration, generally heading WSW. Her final location (a previous short-term stopover) came from along a road, which was later visited and found to be a cattle pond. The reason for the loss of transmissions is unknown for either bird.

## Discussion

This report is the first description of a migratory route for the Orinoco Goose, one of very few intra-Amazonian migrant birds to be studied. A pair of birds tagged in tropical moist rainforest in Manú National Park, Peru migrated to the Llanos de Moxos, Bolivia, a region of savannas, rivers and thousands of lakes. The Llanos de Moxos supports a breeding population of the Orinoco Goose, so birds migrating to Peru appear to be partial migrants.

Partial migration may be central to the evolution of migratory behaviour, providing a transition between sedentariness and complete migration [2]. While most migratory movements, including partial migration, are considered to result from seasonal food scarcity [11], food limitation does not readily explain the migration of herbivorous geese away from a savanna region dotted with lakes and rivers. Large trees with suitable nesting cavities are scarce in the savannah/wetlands of the Llanos de Moxos, occurring only in island patches and along gallery forests [7]. We therefore surmise that some individuals of the Llanos population migrate in response to cavity nest limitation.

The Orinoco Goose migration is notable for its extensive change in longitude relative to latitude. Other birds' migrations include a large longitudinal change, such as the tundra swan [12], and the Northern Wheatear [13]; however, these migrations contain a significant latitudinal component that the Orinoco Goose migration lacks. This directionality may prove to be common for intra-Amazonian migrants from the Manú park, especially given its location near the base of the Andes; however, at this time, too little is known about other migratory species to comment on regional patterns. Being an herbivore, the case of the Orinoco Goose may be distinct.

This study demonstrates that Orinoco Geese migrate at night, both with and without benefit of moonlight and generally follow a compass direction of about 106° while flying overland. These observations suggest that the geese are able to use celestial cues [14] or the Earth's magnetic field [15] for navigation.

In the Llanos de Moxos, the birds foraged in small patches of habitat as indicated by low MCP values. These findings confirm observations of Kriese [4], who showed that Orinoco Geese in Venezuela are highly selective of grazing habitats, preferring the rarest habitat in the landscape, “cespitoso” over all others. Kriese suggested that geese and capybara may maintain small grazing lawns with enhanced nutritive value from repeated clipping, compensatory growth, and repeated fertilization [16]. Although geese may be able to create grazing lawns on their own [17], in the Llanos de Moxos, cattle may aid the same process.

By February 17, 2011, both transmitters failed, and given final locations near roads and human habitation, the birds may have been killed by hunters. However, locals claim that Orinoco Geese are not sought by hunters and moreover, the roads in the region were flooded at the time. (M. Herrera, pers. comm.). Regardless of the uncertainty in what caused the loss of both geese to the study after they arrived in the Llanos de Moxos, it is clear that there are dangers to migratory behavior, and thus to the survival of the Peruvian population of Orinoco Geese. Many bird migrations – indeed many long-distance animal migrations generally – are threatened by increasing loss of habitat, degradation at key stopovers, human impediments to migration and climate change [18]–[19]. On the Río Madre de Dios, the geese spent approximately one week along heavily populated river sections near the confluence of the Río Colorado, where there is an influx of illegal gold miners and severe environmental degradation and pollution (visible in Figure 1, south of Nov 24 & Nov 30 points). Conservation of a Peruvian population of Orinoco Geese will require a multi-national effort to protect birds along their migration route and particularly at their migratory and wet-season stopovers.
